# Comparison of vertical bone resorption following various types of autologous block bone grafts

**DOI:** 10.1186/s40902-023-00406-5

**Published:** 2023-10-17

**Authors:** Hyejin Koo, Junghye Hwang, Byung-Joon Choi, Jung-Woo Lee, Joo-Young Ohe, Junho Jung

**Affiliations:** Department of Oral and Maxillofacial Surgery, School of Dentistry, Kyung Hee University, 26, Kyungheedae-ro, Dongdaemun-gu, Seoul, 02447 Republic of Korea

**Keywords:** Block bone graft, Autogenous bone block, Ramus bone, Chin bone, Iliac bone, Vertical augmentation

## Abstract

**Background:**

This study aims to measure and compare the differences in vertical bone resorption after vertical augmentation using different types of autologous block bone.

**Methods:**

Data were collected from 38 patients who had undergone vertical ridge augmentation using an autologous block bone before implant insertion. The patients were divided into three groups based on the donor sites: ramus bone (RB), chin bone (CB), and iliac crestal bone (IB).

**Results:**

The surgical outcome of the augmentation was evaluated at the follow-up periods up to 60 months. In 38 patients, the mean amount of vertical bone gain was 8.36 ± 1.51 mm in the IB group, followed by the RB group (4.17 ± 1.31 mm) and the CB group (3.44 ± 1.08 mm). There is a significant difference in vertical bone resorption between the groups (*p* < 0.001), and the RB group demonstrated significantly lower resorption than the CB and IB groups (*p* = 0.011 and *p* < 0.001, respectively). The most common postoperative complications included neurosensory disturbance in the CB graft and gait disturbance in the IB graft. Out of the 92 implants inserted after augmentation, four implants were lost during the study period, resulting in an implant success rate of 95.65%.

**Conclusions:**

The RB graft might be the most suitable option for vertical augmentation in terms of maintaining postoperative vertical height and reducing morbidity, although the initial gain was greater with the IB graft compared to other block bones.

## Background

Severe alveolar bone loss result from factors such as tumor excision, advanced periodontitis, or an extended interval following tooth extraction. Successful dental implantation depends on having sufficient high-quality bone at the recipient site [1–3].

Among many bone graft materials, autogenous bone grafts are often considered for vertical augmentation due to their potential for osteogenesis, osteoinduction, and osteoconduction [4, 5]. Moreover, autogenous bone grafts are known for lacking immune rejection and having good biocompatibility, facilitating a rapid healing process [6]. However, they also have certain disadvantages, including a secondary surgical site requirement and a limited amount of available donor bone [6, 7]. Moreover, postoperative complications on the donor or recipient site, temporary neurosensory alterations, infection, ischemia, and dehiscence may occur at the block bone graft site.

There are several donor sites for autologous block bone grafts, including the iliac crest bone (IB), ramus bone (RB), and chin bone (CB). RB grafts are highly beneficial for augmenting atrophic alveolar ridges in partially edentulous patients and exhibit lower resorption rates due to their characteristics as endosteal bone, composed of almost cortical bone [8]. However, there are some disadvantages and precautions associated with their use. These include the risk of inferior alveolar nerve damage, adjacent tooth injury, infection, temporary limitation of mouth opening, and lingual nerve injury resulting from wide or incorrect lingual incisions [8, 9].

CB grafts provide easy access to the surgical site and compose cortical bone with dense bone marrow, resulting in expected increased vascularization after grafting [10]. However, caution must be exercised to avoid vertical incisions near the mental nerve and neurosensory disturbance in the anterior tooth [8, 9, 11].

In cases requiring a significant amount of block bone, IB grafts are often the preferred choice for the recipient site [12]. IB allows for the harvest in larger quantities than RB or CB. IB grafts are also known to have various reported complications, including infection, gait disturbance, and hernia [13, 14]. Moreover, significant surface resorption of transferred IB grafts on the recipient alveolar bone has been observed in many interventions [15, 16].

While many reports have focused on comparisons between block bone and particulate bone graft materials or between two different autogenous block bone grafts, few studies have explored comparisons among three block bone types. This study compares vertical alveolar bone resorption when using various block bone types for grafting to select the most suitable block bone for each case. This approach aims to achieve sufficient bone width and height, facilitating oral rehabilitation during the placement of dental implants in atrophic alveolar ridges.

## Methods

A retrospective analysis was conducted on patients with vertical augmentation before implant placement using autologous block bone at the Department of Oral & Maxillofacial Surgery, Kyung Hee University Dental Hospital from 2010 to 2021. The block bone donor sites used were RB, CB, and IB. The criteria for selecting the RB and CB groups in the randomized trial were determined based on patient and operator preferences. IB grafts were chosen when vertical bone loss exceeded 5 mm. Patients aged 17 years and older classified as ASA I or II were included in this study. Oncology patients previously undergoing radiotherapy in the head and neck region were excluded. Patients with a follow-up period of less than 12 months after the block bone graft or without radiographic records were excluded. Patients who only received block bone grafts without implant placement, underwent horizontal augmentation, or received crushed bone grafts were also excluded. Of the eligible patients, 38 met the selection criteria and were included in this study. Demographic data, including medical histories, dates of the graft surgery, locations of bone grafts, types of autogenous bone grafts used, additional bone graft materials employed, and type of implants, were obtained by reviewing patient medical records. All protocols were approved by the Institutional Review Board (IRB) of Kyung Hee University Dental Hospital (KH-DT23018).

### Surgical procedures

**Fig. 1:**
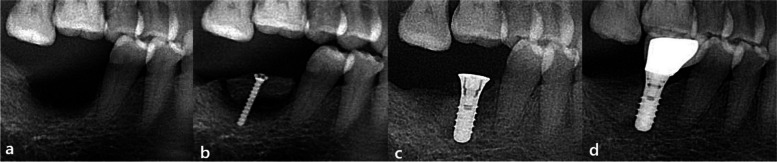
Block bone graft and implant insertion. **a** Before grafting, partial edentulous state on the right mandibular molar region (I). **b** Right after vertical augmentation of RB (T0). **c** After 4 to 8 months, implants were placed and the crew was removed. **d** Thirty-six months after grafting (T4), the final prosthetic was completed

In all three groups, the surgical procedures consisted of two stages: vertical augmentation with autologous block bone surgery and implant placement surgery. The implant placement was performed after a healing period of 4 to 8 months following the bone augmentation (Fig. 1).

In the IB group, both the harvesting and augmentation of the IB were carried out under general anesthesia in the operation room. Approximately 3–4 days later, postoperative radiographic images were taken to assess the surgical outcome.

The surgeries were performed under local anesthesia for the RB graft and CB graft groups. Each block bone was harvested according to the surgical plan. After the procedure, all patients underwent postoperative radiographic images.

All patients were prescribed postoperative antibiotics and non-steroidal anti-inflammatory drugs (NSAIDs) to ensure proper control and prevention of infection, bleeding, and pain. Patients were instructed to maintain meticulous plaque control and follow a soft diet for an additional one to 2 weeks. Sutures were typically removed after an average of 10 days.

### Radiographic evaluation and measurement of the height of bone change

**Fig. 2:**
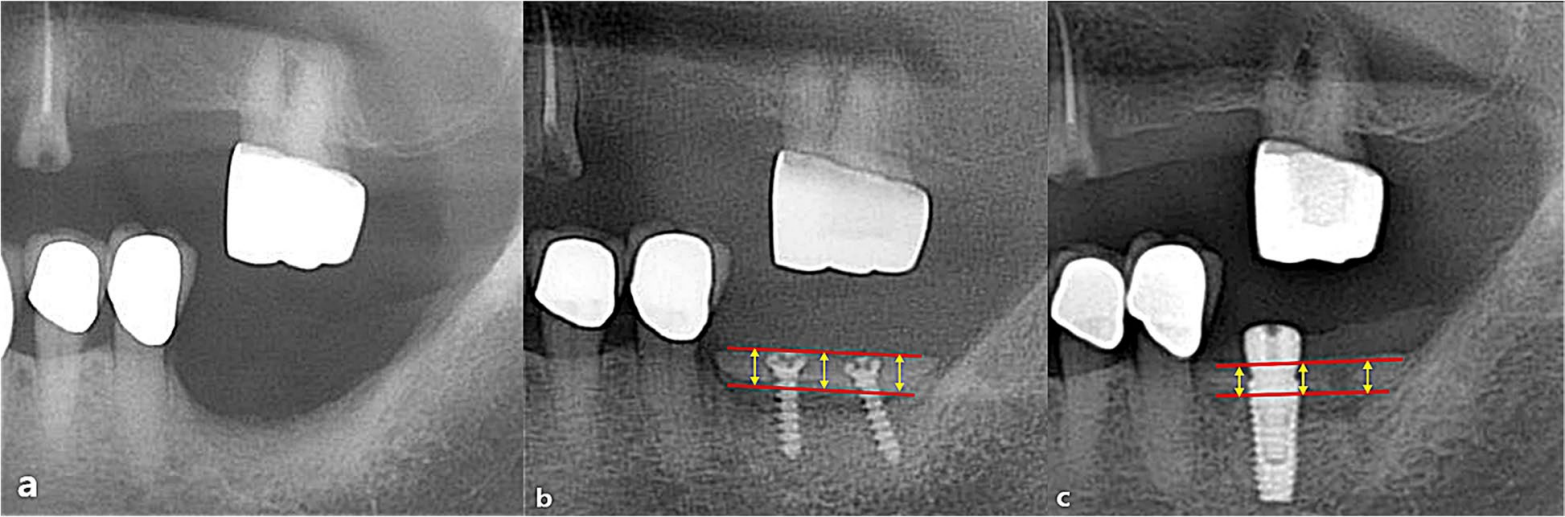
Measurement of the vertical amount of bone gain and resorption amount. **a** Vertical alveolar bone gain was measured using an immediate post-operative radiograph, calculating the distance between the adjacent tooth and the top of the grafted bone or the head of the screw. **b** Three months after augmentation, the extent of vertical bone resorption was assessed

To analyze the vertical bone loss and compare the three types of autologous block bone grafts, a 50 × 50 grid was superimposed on pre and postoperative radiographic images of each patient during specific periods. Plain panoramic views were the primary imaging method used (ASAHI Roentgen® parameters of 72 ~ 76 kVp, 8 mA), and periapical views (Dentsply sirona®, parameters of 60 kVp, 6 mA, and exposure time of 0.16 ~ 0.20 s) were employed as supplementary imaging (Fig. 2). The time periods were categorized as follows: before bone augmentation (I), immediately after bone augmentation (T0), 3 months after bone augmentation (T1), 6 months after bone augmentation (T2), 12 months after bone augmentation (T3), 36 months after bone augmentation (T4), and 60 months after bone augmentation (T5). The average marginal bone height was measured and recorded for each time period. To ensure accuracy, the bone height was calculated twice to eliminate any potential statistical errors. All measurements were conducted using the PACS calibration system (PiView-Star®, version 5.0.1, Infinitt Co., Seoul, Korea).

### Vertical bone gain

The amount of vertical bone gain was assessed by measuring the distance between the uppermost and lowermost margins of the grafted block bone at the mesial, middle, and distal regions, with the adjacent tooth serving as the reference point (Fig. 2). Subsequently, the average value was calculated. To ensure reliability, the initial preoperative bone height was measured twice, yielding an intraclass correlation coefficient of 0.958.

### Vertical bone resorption

The distance between the uppermost point of the block bone and the implant shoulder (or the top of the screw head) at the mesial, middle, and distal regions were measured at each time period, and the average value was calculated.

After the insertion of dental implants, the magnification factor was determined based on the length of the implant fixture. This magnification factor was then applied to each patient, and a correction value was obtained by dividing the magnification factor by the actual measurement value.

### Evaluation of implant survival and success rates

Implant survival was defined as the presence of the implant at the end of the follow-up period [17]. Implant success was determined based on the criteria by Albrektsson and colleagues, which include “bone loss of less than 0.2 mm annually after the implant’s first year of service, absence of peri-implant radiolucency, and the absence of persistent pain, discomfort, or infection.”

### Statistical analysis

A linear mixed model (LMM) was utilized to assess differences among three groups overtime periods. We conducted Mann-Whitney *U* tests to compare two groups at each time point. These analyses were conducted using SPSS software (version 25.0, Chicago, IL). Logistic regression analysis was conducted to analyze the factors associated with vertical bone resorption. *p* values less than 0.05 were considered statistically significant.

## Results

This study included 38 patients with 47 block bone graft procedures and received a total of 92 implants. Among them, 13 patients (mean age 57.62, *M*:*F* = 4:9) underwent RB, 13 patients (mean age 34.69, *M*:*F* = 5:8) underwent CB, 12 patients (mean age 53.92, *M*:*F* = 4:8) underwent IB procedures, respectively. All patients had implants at the grafted sites within 4 to 8 months after block bone grafting. The total follow-up period averaged 43 months (13 to 76 months) (Table 1).

**Table 1:** Demographic data

	**RB**	**CB**	**IB**
**No. of patients (M:F)**	13 (4:9)	13 (5:8)	12 (4:8)
**Age (years)**	57.62 ± 11.96	34.69 ± 16.64	53.92 ± 13.94
**Mean follow-up period**	49 months	35 months	46 months
**No. of implants**			
**Maxilla posterior (right:left)**	4 (1:3)	2 (0:2)	13 (7:6)
**Mandible posterior (right:left)**	17 (9:8)	3 (3:0)	25 (10:15)
**Maxilla anterior (right:left)**	1 (1:0)	11 (5:6)	3 (2:1)
**Mandible anterior (right:left)**	0 (0:0)	9 (3:6)	4 (2:2)
**Total**	22	25	45
**Utilization and types of additional bone graft material per block bone**			
**Xenograft**	5	3	3
**Allograft**	5	0	2
**Xenograft + allograft**	3	2	0
**Synthetic**	1	0	0
**None**	0	8	15
**Total**	14	13	20

The greatest vertical augmentation was observed in the IB group, measuring 8.36 ± 1.51 mm, followed by the RB group and CB group with 4.17 ± 1.31 mm and 3.44 ± 1.08 mm, respectively. In the RB group, bone resorption was observed continuously at 3, 6, 12, 36, and 60 months after augmentation, and the remaining bone height was 3.74 mm, 3.58 mm, 3.37 mm, 2.97 mm, and 2.77 mm, respectively. Similarly, in the CB group, bone resorption persisted with the remaining bone height of 3.03 mm, 2.93 mm, 2.55 mm, 2.28 mm, and 1.73 mm. In the IB group, bone resorption also continued with the highest resorption, and the remaining bone height was measured as 7.25 mm, 6.59 mm, 5.66 mm, 4.62 mm, and 3.66 mm (Table 2). Most graft material resorption occurred within 12 months after augmentation, with a measurement of 0.80 mm in the RB group, 0.89 mm in the CB group, and the IB group 2.80 mm at 12 months postoperation. The majority of dental implants were placed in the grafted sites between 4 and 8 months.

**Table 2:** Vertical bone resorption at each period (mm) and comparison between groups

	**The mean amount of augmentation (mean ± SD, mm)**	**The mean amount of vertical bone resorption after grafting (mean ± SD, mm)**				
		3 Mo (T0-T1)	6 Mo (T0-T2)	12 Mo (T0-T3)	36 Mo (T0-T4)	60 Mo (T0-T5)
**RB**	4.17 ± 1.31	0.33 ± 0.15	0.59 ± 0.25	0.80 ± 0.31	1.20 ± 0.47	1.40 ± 0.17
**CB**	3.44 ± 1.08	0.36 ± 0.24	0.51 ± 0.34	0.89 ± 0.53	1.16 ± 0.23	1.71 ± 0.35
**IB**	8.36 ± 1.51	1.11 ± 0.40	1.77 ± 0.74	2.80 ± 0.95	3.74 ± 0.88	4.70 ± 0.90
**Comparison between groups at each time point (*p* value)**						
**RB-CB**		0.748	0.437	0.430	0.841	0.400
**RB-IB**		< 0.001	< 0.001	< 0.001	< 0.001	0.002
**CB-IB**		< 0.001	< 0.001	< 0.001	< 0.001	0.002
**Comparison between groups over the entire follow-up period (*p* value)**						
**RB-CB**		0.011				
**RB-IB**		< 0.001				
**CB-IB**		< 0.001				

*p* < 0.05 was considered as statistical significance

*RB* Ramus bone, *CB* Chin bone, *IB* Iliac bone

**Fig. 3:**
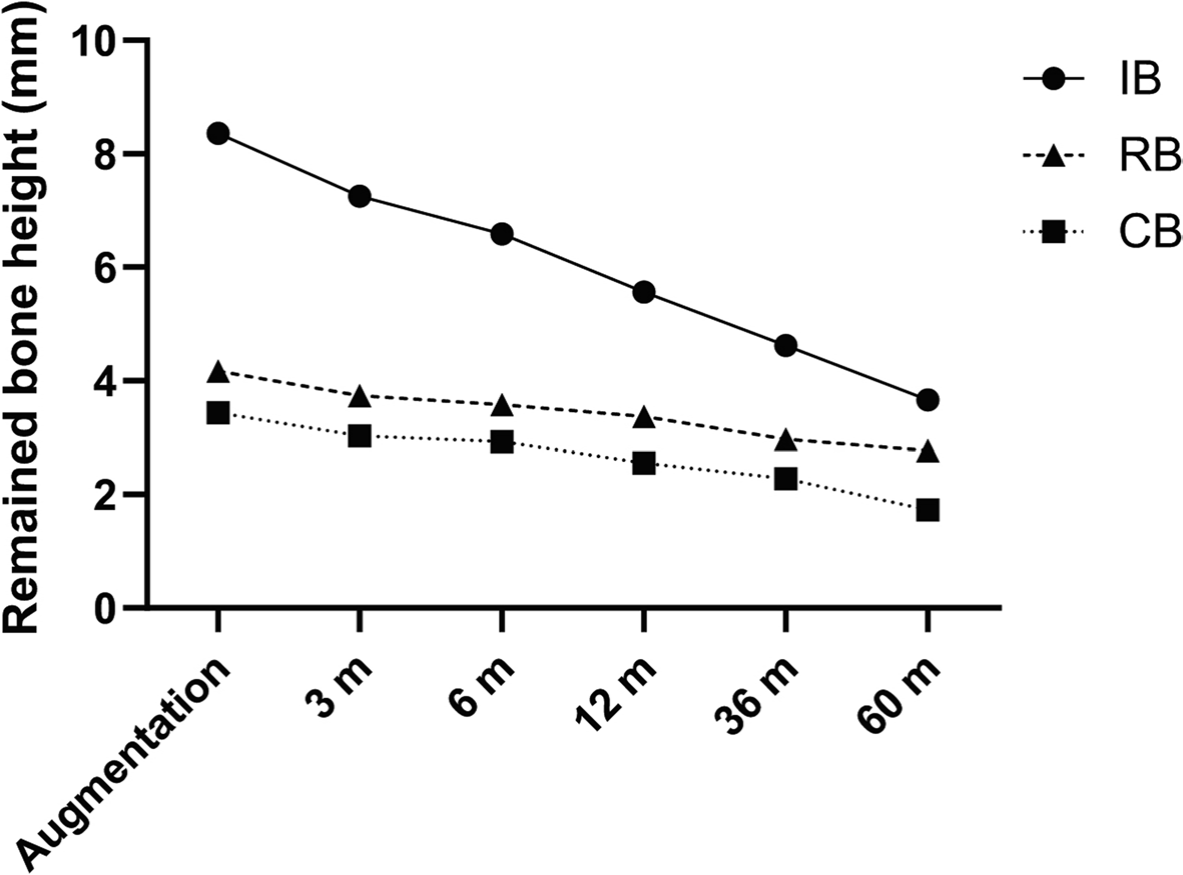
Changes in the height of augmented bone

Over the entire study period, consistent vertical bone resorption was observed in the augmented bone (Fig. 3), and a significant difference was found among the three groups (*p* < 0.001). The vertical bone resorption in the RB group was significantly lower than those in the CB and IB groups when accounting for the entire follow-up period (*p* = 0.011 and *p* < 0.001, respectively; Table 2). However, the vertical resorption was not significantly different between the RB and CB groups when comparing at each time point. In contrast, both RB and CB groups showed significantly lesser vertical bone resorption than the IB group at all time points (*p* < 0.001) (Table 2).

### Factors associated with vertical bone resorption

Out of the 92 implants, 14 implants did not meet the criteria for implant success, resulting in a success rate of 85%. Specifically, the RB and CB groups each had two implants that did not meet the criteria, while the IB group had ten implants that did not fulfill the success criteria. However, it was not statistically related to vertical bone resorption (*p* = 0.762). Notably, among these 14 implants, four implants inserted in the IB group were eventually lost, and the overall survival rate in this study was 95.65%. Out of the lost implants, three implants from a patient who underwent implant surgery on the left mandibular molar area were removed at 15 months and 4 and 6 years due to peri-implantitis accompanied by block bone resorption. The only factor found to be related to vertical bone resorption was the type of block bone graft, specifically IB (*p* = 0.002; Table 3).

**Table 3:** Analysis of factors associated with vertical bone resorption

	***p* value**	**Exp(B)**
**Age**	0.574	1.025
**Gender**	0.789	0.484
**Bone metabolic disease**	0.994	0.981
**DM**	0.712	0.484
**Smoking**	0.133	12.449
**Implant survival**	0.102	12.570
**Implant success**	0.762	0.687
**Block bone type**		
**RB**	Ref	Ref
**CB**	0.377	5.351
**IB**	0.002	275.288

*p* < 0.05 was considered as statistical significance

*DM* Diabetes mellitus, *RB* Ramus bone, *CB* Chin bone, *IB* Iliac bone

### Postoperative complications

Intraoperative complications were rarely reported in all groups. However, the most common postoperative complications in the IB graft groups were relatively frequent. Gait disturbance was a distinctive postoperative complication in the IB group, followed by wound dehiscence, ecchymosis, and bleeding at the recipient site. Neurosensory alterations in the mandibular incisor teeth were reported by 3 patients in the CB group. The RB group exhibited the lowest incidence of postoperative complications (Table 4).

**Table 4:** Postoperative complications

	**RB (no. of patients)**	**CB (no. of patients)**	**IB (no. of patients)**
**Swelling and pain**	0	2	0
**Bleeding on the recipient site**	0	0	1
**Neurosensory alterations**	0	3	0
**Gait disturbance**	0	0	7
**Discomfort on adjacent teeth**	1	0	0
**Hematoma, ecchymosis on donor site**	0	0	1
**Infection (pus discharge)**	1	2	0
**Wound dehiscence**	0	1	2
**Ulcer**	0	0	1

## Discussion

Autogenous bone graft offers several advantages, including the absence of foreign body reactions, reduced risk of infection, better predictability, and favorable soft tissue reaction. Despite the inconvenience of the donor site, it remains the gold standard to this day. Volumetric stability of graft is a critical factor for implant survival and success rates, which can be achieved through sufficient bone quantity and quality [12, 18, 19]. This study aimed to evaluate the differences in vertical bone resorption following three types of autologous block bone augmentation, namely RB, CB, and IB. To date, only a limited number of studies have comprehensively analyzed RB, CB, and IB grafts simultaneously.

Several studies have reported a relatively high and significant initial vertical resorption in IB grafts during the first year following autologous bone grafting, which is consistent with the findings of this study [20, 21]. Additionally, various clinical studies suggested that intraoral bone grafts have more cortical bone and smaller trabecular bone than IB grafts. These characteristics enhance integration with the recipient site, improve the recipient site’s survival capacity, and promote better volume maintenance [22–28]. Consequently, IB grafts tend to exhibit a higher graft resorption rate, while CB or RB grafts have shown lower resorption rates during the initial postoperative period [29]. However, the relatively higher trabecular structure in IB grafts might lead to faster healing and resistance to local infections compared to RB or CB grafts [30, 31]. Nevertheless, definitive conclusions remain elusive due to the lack of standardization for objective comparisons [30].

In this analysis, IB grafts showed higher resorption rates than the other two graft types, although they resulted in the highest remaining bone volume among the three, due to their initial vertical bone gain. RB graft led to less vertical resorption compared to CB and IB grafts. These findings align with previous comparative studies. However, the interpretation of bone resorption quantities requires further investigation, particularly in relation to simultaneous implant placement with block bone grafts [32].

However, IB grafts were predominantly utilized for long-span augmentation procedures, required for full-arch augmentation, as they facilitated successful vertical and horizontal bone volume augmentation [12]. When autogenous bone is harvested from CB or RB, the quantity that can be collected could be limited, although the bone resorption rate is lower and more predictable compared to that of IB. On the other hand, intraoral block bone grafts may offer the advantage of maintaining bone quality better than extra-oral donor bone [12]. Due to their membranous nature, intraoral bone grafts exhibit less bone resorption after grafting compared to endochondral bone grafts. Additionally, intraoral bone grafts have better revascularized the transplanted tissue, resulting in improved integration with the recipient site. As a result, intraoral bone grafts are recognized for achieving superior osseointegration at the graft site compared to extraoral bone grafts [33–35].

In the current study, intraoral bone grafts showed fewer postoperative complications and lower morbidity compared to IB grafts. IB showed more frequent complications than the other grafts, including distinctive complications such as gait disturbance, wound dehiscence, and ecchymosis at the donor site. The CB group had distinctive complaints about temporary neurosensory disturbance in the anterior mandibular area, although it decreased over time.

In previous studies, the survival rates of implants placed into the autogenous bone block augmentation have ranged from 90.01 to 100% [2], and the success rate has been reported to be between 89.5 and 95.7% [36]. In another previous systematic review, the implant survival rate was 90.4% for autogenous bone grafts, with follow-up periods ranging from 5 to 74 months [37]. In this study, while the survival rate was 95.65%, the success rate was 85%. This difference may be attributed to a greater amount of bone resorption observed in patients with IB grafts.

Due to limitations associated with plain radiographic images, the accuracy of measuring vertical bone height remains questionable. Follow-up CBCT scans are necessary for a more precise evaluation of bone resorption. Additionally, this study did not assess the width of keratinized gingiva, which is important to acknowledge as the presence and the width of keratinized gingiva may potentially influence peri-implant health and crestal bone loss. Reduction of keratinized gingiva is often associated with atrophied alveolar ridges. Further studies with larger sample sizes and rigorous control of confounding factors are imperative to solidify these findings.

## Conclusions

This study assessed the extent of vertical block bone resorption following RB, CB, or IB grafting. Additionally, the stability of implant placement at the grafted sites was evaluated over up to 60 months. RB grafts resulted in less vertical resorption, while IB grafts showed a higher resorption rate compared to the other two types, despite achieving the greatest remaining bone volume among the three, primarily due to their initial vertical gain. RB grafts might be a favorable option for vertical augmentation as they help maintain postoperative vertical height while minimizing surgical morbidity. However, using IB grafts becomes inevitable when a substantial amount of vertical bone is required, provided careful periodontal management, due to the potential for crestal bone loss.

## Data Availability

The datasets used and/or analyzed during the current study are available from the corresponding author on reasonable request.

## Abbreviations

RB: Ramus bone
CB: Chin bone
IB: Iliac crestal bone
CBCT: Cone beam computed tomography
